# Effect of body mass index on pregnancy outcomes in a freeze-all policy: an analysis of 22,043 first autologous frozen-thawed embryo transfer cycles in China

**DOI:** 10.1186/s12916-019-1354-1

**Published:** 2019-06-26

**Authors:** Jie Zhang, Hongfang Liu, Xiaoyan Mao, Qiuju Chen, Yong Fan, Yitao Xiao, Yun Wang, Yanping Kuang

**Affiliations:** Department of Assisted Reproduction, Shanghai Ninth People’s Hospital, Shanghai Jiao Tong University School of Medicine, 639 Zhizaoju Rd, Shanghai, 200011 China

**Keywords:** Frozen-thawed embryo transfer, Body mass index, Reproductive outcomes

## Abstract

**Background:**

Abnormal BMI is associated with discouraging IVF outcomes in fresh autologous or oocyte donor cycles, whether or not such a relation also holds true for women undergoing frozen-thawed embryo transfer (FET) remains unknown. In addition, it remains unclear the detrimental effect of abnormal BMI on IVF outcomes occurs at the level of ovary or endometrium.

**Methods:**

A retrospective study involved 22,043 first FET cycles of all women who had undergone a freeze-all policy during the period from January 2010 to June 2017. To control for the embryo factor, our analysis was restricted to women with high-quality embryo transfer. The main outcome measure was live birth rate per embryo transfer. The secondary endpoints included rates of implantation, clinical pregnancy, multiple pregnancy, and pregnancy loss. Multivariate logistic regression analysis was performed to detect the independent effect of BMI on live birth rate after adjusting for important confounding variables.

**Results:**

In the crude analysis, reproductive outcomes were similar between underweight women and normal-weight controls whereas all parameter outcomes were significantly worse in patients with obesity. After adjustment for a number of confounding factors, underweight women had a marginally significant decrease in rates of implantation (adjusted odds ratio (aOR) 0.91; 95% CI 0.85–0.96), clinical pregnancy (aOR 0.91; 95% CI 0.83–0.99), and live birth (aOR 0.91; 95% CI 0.83–0.99) as compared to the women with normal weight. Obesity was significantly associated with decreased implantation (aOR 0.80; 95% CI 0.73–0.87), clinical pregnancy (aOR 0.81; 95% CI 0.71–0.91), and live birth rates (aOR 0.70; 95% CI 0.62–0.80). Moreover, the pregnancy loss rate, both in the first (aOR 1.46; 95% CI 1.15–1.87) and in the second trimester (aOR 2.76; 95% CI 1.67–4.58), was significantly higher in the obesity group than that in the reference group.

**Conclusions:**

Among women undergoing first FET with high-quality embryo transfer, low BMI has limited impact on pregnancy and live birth rates. On the contrary, obesity was associated with worse IVF outcomes. Our findings further highlighted that endometrial receptivity played an important role in the poor reproductive outcomes of women with abnormal weight status.

**Electronic supplementary material:**

The online version of this article (10.1186/s12916-019-1354-1) contains supplementary material, which is available to authorized users.

## Background

Obesity prevalence is on the rise at an alarming rate. More than 1.9 billion adults worldwide were overweight in 2016 and of these 650 million were obese. According to the World Health Organization statistics, in Europe, an estimated 54.3% of women were overweight and obesity afflicted approximately one quarter of women with reproductive age [1].

It is widely accepted that elevated body mass index (BMI) exerts detrimental effects on female fecundity [2]. Obesity could affect nearly all the components of reproductive system, ranging from oocyte competence over embryo quality to uterine environment [3]. In natural conception cycles, the chance of a spontaneous pregnancy declined linearly as a BMI over 29 kg/m^2^ [4]. Furthermore, a matched case-control study showed that obesity was associated with increased risk of first trimester and recurrent miscarriage [5]. With IVF treatment, women with elevated BMI had significantly lower clinical pregnancy and live birth rates and had a significantly higher miscarriage rate as compared to normal controls [6].

The mechanism by which obesity impairs reproductive potential is far less clear. Over the past decade, ongoing debate continues regarding whether the adverse impact of increased BMI on IVF outcomes acts at the level of ovary or endometrium. A number of studies found an inverse relationship between BMI and oocyte or embryo quality [7, 8]. While others claimed such an association was not observed, instead, pointed out that endometrial dysfunction is the major player in the poor reproductive performance of obese women [9, 10]. Existing literature on this subject mainly focused on autologous fresh or oocyte donor cycles. Data on fresh autologous cycles failed to discern the actual role of oocyte and endometrium due to the fact that the hormonal milieu generated from controlled ovarian stimulation during fresh IVF might have biased the results [11]. The ovum donation model provides an invaluable tool to isolate the effect of endometrium. Nonetheless, a systematic review and meta-analysis of donor oocyte cycles showed the pregnancy outcomes in obese recipients were not inferior to those with normal BMI range, further suggesting that oocyte quality rather than endometrial receptivity was the culprit for negative IVF outcomes in obese women [12].

Frozen-thawed embryo transfer (FET), which allows for transfer of embryos into a more physiologic uterine environment, has attracted much attention in recent years [13]. Very few studies have been designed to evaluate the impact of BMI on FET cycles. A more recent study, including 461 women who underwent an identical freeze/thaw protocol with high-quality blastocysts transfer, was unable to find an association between BMI and the chance of implantation, pregnancy, or live birth. Nonetheless, this study was limited by a small sample size, with merely 59 women in the obesity group and 9 women in the underweight group [14]. Furthermore, inadequate selection criteria prevented it from drawing solid conclusions. With the increasing use of FET worldwide, exploration of topic on the influence of BMI on reproductive outcomes resulting from FET cycles is of vital importance.

Thus, the aim of the current study is to identify the effect of BMI on pregnancy outcomes in a large cohort of women undergoing first FET cycles. Attempts to control for ovarian factors, only patients with high-quality embryo transfer were included.

## Methods

### Study design and population

A retrospective study was conducted at the Department of Assisted Reproduction of the Ninth People’s Hospital of Shanghai Jiao Tong University School of Medicine. Women who had undergone first FET cycles with high-quality embryo transfer (described below) after a freeze-all policy during the period from January 2010 to June 2017 were enrolled. Exclusion criteria were as follows: patients older than 40 years of age; a history of recurrent miscarriage (defined as ≥ 2 previous biochemical/ clinical losses); previous IVF attempts regardless of a prior fresh or frozen embryo transfer; the presence of submucosal fibroids or polyps, intramural fibroids > 4 cm, hydrosalpinx, and congenital uterine malformation as determined by three-dimensional ultrasound and hysterosalpingography. Patients with hypertension, diabetes, or thyroid dysfunction were also excluded.

Patients were categorized into four groups according to Asian BMI classification [15], namely underweight (BMI < 18.5 kg/m^2^), normal weight (18.5–22.9 kg/m^2^), overweight (23–27.4 kg/m^2^), and obese (BMI ≥ 27.5 kg/m^2^). BMI was measured by a trained nurse at the start of treatment initiation. This study protocol was approved by the ethical committee of the hospital (reference number 2017-211) and was carried out in accordance with the Helsinki Declaration. Due to the retrospective nature, informed consent was not required and patients’ data were used anonymously.

### Endometrial preparation prior to embryo transfer

Modified natural cycles were applied for patients with regular ovulatory cycles, in which ultrasound monitoring was initiated on days 10–12 of the menstrual cycle. When the dominant follicle reached a mean diameter of ≥ 17 mm and endometrial thickness attained ≥ 7 mm, with estradiol > 150 pg/ml and *P* < 1 ng/ml, the timing of hCG triggering depended on the occurrence of an LH surge. On detection of a serum LH surge (LH ≥ 20 IU/l and more than double the average LH level over the past 2 days), a bolus of urinary hCG (5000 IU; Lizhu Pharmaceutical Trading Co.) was injected in the same afternoon. Exogenous progesterone (400 mg/day; Utrogestan; Besins Healthcare) was given vaginally which started 2 days after hCG administration and day 3 embryo transfer was scheduled 4 days later (6 days later for blastocyst transfer). In the absence of a LH surge (LH < 20 IU/l), hCG was injected at 9:00 p.m. and embryo transfer was arranged 5 days later for 3-day-old embryos or 7 days later for blastocysts. Luteal support was initiated 3 days after hCG triggering.

In patients with irregular menses, endometrial preparation was performed in either a mildly letrozole-stimulated cycle or an artificial cycle (AC), depending on patients’ preference and the discretion of treating physicians. In letrozole-FET cycles, letrozole (Hengrui Medicine Co, Jiangsu, China) was prescribed orally for 5 days initiating on cycle day 3 of spontaneous menses or progesterone-induced withdrawal bleeding, at a daily dose of 5 mg. Ultrasound monitoring and serum hormone analysis were performed from cycle day 10 onwards. If the leading follicle reached a diameter of ≥ 14 mm on cycle day 10, transvaginal ultrasound was repeated every 2 days and no other drugs were added until ovulation triggering. In case of a dominant follicle < 14 mm on day 10, a daily dosage of 75 IU of hMG (Anhui Fengyuan Pharmaceutical Co.) was supplemented to stimulate follicle growth, with incremental doses of 37.5 IU if needed. The timing of ovulation triggering, FET scheduling, and luteal support was the same as above described in natural-FET cycles. In AC-FET cycles, oral 17β-estradiol (Fematon 2 mg, three times daily; Abbott Healthcare Products B.V.) was commenced on the second or third day of a natural or progesterone-induced menstrual cycle. When the endometrial thickness attained ≥ 7 mm, progesterone exposure was initiated. Embryo transfer was performed 3 days after progesterone administration for day 3 embryos or 5 days later for blastocysts. In all study groups, luteal support was continued to 10 weeks of gestation if a pregnancy occurred.

### Embryo quality assessment and vitrification

Embryo morphology assessment was evaluated on day 3, day 5, and day 6. Cleavage-stage embryos with at least 7 blastomeres and fragmentation < 20% were regarded as high-quality embryos [16]. Blastocysts were scored according to the Gardner and Schoolcraft grading system [17] and recorded as high quality if they reached at least an expansion stage 3 with A or B for inner cell mass and trophectoderm (3BB). In all FET cycle, no more than 2 embryos can be transferred. Of note, during the study period, a complete change in the type of culture medium was made. Specifically, before January 2013, commercially sequential available culture medium (Irvine Scientific) was used. From January 2013 onwards, continuous single culture media (Irvine Scientific) was introduced. Except for this switch, the other laboratory procedures and conditions remained constant.

The vitrification and thawing procedure was previously described by Kuwayama et al. [18]. Briefly, embryo vitrification was carried out via a Cyrotop carrier system, in conjunction with DMSO-EG-S as cryoprotectants. For thawing, embryos were transferred into dilution solution in a sequential manner (1 M–0.5 M–0 M sucrose).

### Outcome parameters and statistical methods

The primary outcome of the study was the live birth rate. Secondary endpoints included rates of implantation, clinical pregnancy, multiple pregnancy, miscarriage, and perinatal outcomes.

Live birth was defined as a live neonate born after 24 weeks of gestation. Ongoing pregnancy was defined as a viable pregnancy that progressed beyond 12 weeks’ gestation. A clinical pregnancy was confirmed by the observation of a gestational sac on ultrasound scanning 5 weeks after embryo transfer. Miscarriage rate was defined as a loss of clinical pregnancy before the 24th gestational week. The implantation rate was calculated as the number of gestational sacs visualized on ultrasound examination divided by the number of embryos transferred. All calculations were made on a “per embryo transfer” basis. Low birth weight and macrosomia were defined as birth weights < 2500 g and ≥ 4000 g, respectively. Preterm birth was defined as a delivery before complete 37 gestational weeks. Pregnancy-related complications included gestational diabetes, pregnancy-induced hypertension, and pre-eclampsia. All neonatal and delivery information was obtained from electronic medical records.

The baseline characteristics and associated clinical outcomes were compared via *t* test or chi-square test where appropriate. A single multivariate logistic regression analysis, incorporating the BMI group as a categorical explanatory variable, was performed to determine the independent effect of BMI on reproductive outcomes after adjustment for possible confounding factors, including maternal age, infertility duration, obstetric history (gravidity and parity), infertility cause, number of ovum pick-up (OPU) cycles prior to FET, year of FET, number of embryo transferred, embryo stage at transfer, and embryo quality. Normal-weight women were used as the referent group for all comparisons. All statistical analyses were performed with the use of the Statistical Package for Social Sciences (SPSS) version 21.0. A *P* value of < 0.05 was considered to be statistically significant.

## Results

A total of 22,043 patients met the inclusion criteria and underwent first FET cycles during the study period. Mean BMI was 21.75 ± 3.12 kg/m^2^, with 11.5% underweight, 60% normal, 23% overweight, and 5.5% obese. A flow diagram of the patient selection process is shown in Additional file 2: Figure S1. Baseline characteristics across BMI groups are presented in Table 1. Women with normal weight (31.36 ± 3.73 years) were slightly older than underweight (30.56 ± 3.61 years) and obese subjects (31. 11 ± 3.79 years) and were slightly younger than overweight subjects (31.66 ± 3.79 years). Overweight and obese women had significantly longer infertility duration than normal controls. The prevalence of PCOS increased with increasing BMI, while the rates of endometriosis and diminished ovarian reserve decreased. No difference was seen in insemination method across all BMI categories. Compared with normal-weight patients, women in the overweight and obesity categories more frequently experienced more than one ovum pick-up (OPU) cycle prior to FET.

**Table 1:** Characteristics of the FET cycles for each study group

Characteristics	Underweight (< 18.5 kg/m^2^)	Normal weight (18.5–22.9 kg/m^2^)	Overweight (23–27.4 kg/m^2^)	Obese (≥ 27.5 kg/m^2^)	*P* value^a^	*P* value^b^	*P* value^c^
Patients, *n*	2527	13,224	5079	1213			
Age (years)	30.56 ± 3.61	31.36 ± 3.73	31.66 ± 3.79	31.11 ± 3.79	< 0.001	< 0.001	0.023
BMI (kg/m^2^)	17.66 ± 0.73	20.67 ± 1.21	24.68 ± 1.22	29.89 ± 2.39	< 0.001	< 0.001	< 0.001
Infertility duration (years)	3.19 ± 2.37	3.36 ± 2.75	3.68 ± 2.90	4.10 ± 2.97	0.004	< 0.001	< 0.001
Gravidity					< 0.001	0.121	0.448
0	1577(62.4)	7393(55.9)	2756(54.3)	672(55.4)			
1	556(22.0)	3042(23.0)	1199(23.6)	297(24.5)			
≥ 2	394(15.6)	2789(21.1)	1124(22.1)	244(20.1)			
Parity					< 0.001	0.002	0.520
0	2418(95.7)	12,220(92.4)	4614(90.8)	1120(92.3)			
1	105(4.2)	938(7.1)	429(8.5)	84(6.9)			
≥ 2	4(0.2)	66(0.5)	36(0.7)	9(0.7)			
Main cause of infertility					0.007	< 0.001	< 0.001
Tubal factor	1341(53.1)	7198(54.4)	2666(52.5)	553(45.6)			
PCOS	162(6.4)	1077(8.1)	796(15.7)	346(28.5)			
Diminish ovarian reserve	183(7.2)	875(6.6)	278(5.5)	41(3.4)			
Endometriosis	261(10.3)	1179(8.9)	307(6.0)	44(3.6)			
Uterine factor	75(3.0)	426(3.2)	175(3.4)	26(2.1)			
Unexplained	94(3.7)	424(3.2)	136(2.7)	46(3.8)			
Male factor	411(16.3)	2045(15.5)	721(14.2)	157(13.9)			
Number of OPU prior to FET					0.043	< 0.001	< 0.001
1	1962(77.6)	10,028(75.8)	3689(72.6)	824(67.9)			
2	338(13.4)	2026(15.3)	895(17.6)	241(19.9)			
≥ 3	227(9.0)	1170(8.8)	495(9.7)	148(12.2)			
Fertilization method					0.744	0.789	0.584
IVF	1605(63.5)	8470(64.1)	3252(64.0)	795(65.5)			
ICSI	601(23.8)	3145(23.8)	1225(24.1)	277(22.8)			
IVF+ ICSI	321(12.7)	1609(12.2)	602(11.9)	141(11.6)			
Year of treatment					0.659	< 0.001	0.037
2010–2012	550(21.8)	2836(21.4)	932(18.4)	222(18.3)			
2013–2014	881(34.9)	4736(35.8)	1809(35.6)	453(37.3)			
2015–2017	1096(43.4)	5652(42.7)	2338(46.0)	538(44.4)			

^a^Underweight vs. normal weight

^b^Overweight vs. normal weight

^c^Obese vs. normal weight

Data are presented as mean ± SD for continuous variables and *n* (%) for dichotomous variables

Detailed information on the FET cycles is summarized in Table 2. There were no statistically significant differences in the number of embryo transfer, embryo quality, and endometrial thickness on the day of embryo transfer among groups. However, in terms of the type of endometrial preparation, more mildly stimulated and substituted cycles were performed in women who are overweight and obese than those in normal controls (both *P* < 0.001).

**Table 2:** Cycle characteristics of FET

Characteristics	Underweight (< 18.5 kg/m^2^)	Normal weight (18.5–22.9 kg/m^2^)	Overweight (23–27.4 kg/m^2^)	Obese (≥ 27.5 kg/m^2^)	*P* value^a^	*P* value^b^	*P* value^c^
Patients, *n*	2527	13,224	5079	1213			
Endometrial preparation					0.400	< 0.001	< 0.001
Modified Natural cycle	1202(47.6)	6104(46.2)	1961(38.6)	356(29.3)			
Stimulated cycles	655(25.9)	3479(26.3)	1588(31.3)	434(35.8)			
Hormonal replacement	670(26.5)	3641(27.5)	1530(30.1)	423(34.9)			
Number of embryo transferred					0.913	0.457	0.124
1	264(10.4)	1372(10.4)	508(10.0)	143(11.8)			
2	2263(89.6)	11,852(89.6)	4571(90.0)	1070(88.2)			
Embryo developmental stage at transfer					0.481	< 0.001	0.007
Day 3	2382(94.3)	12,417(93.9)	4843(95.4)	1162(95.8)			
Day 5/6	145(5.7)	807(6.1)	236(4.6)	51(4.2)			
Embryo quality					0.201	0.584	0.158
Top quality	553(21.9)	3048(23.0)	1190(23.4)	258(21.3)			
Good quality	1974(78.1)	10,176(77.0)	3889(76.6)	955(78.7)			
Post-thaw embryo survival rate	4790/4812 (99.54)	25,076/25,236 (99.37)	9650/9707 (99.41)	2283/2298 (99.35)	0.147	0.618	0.914
Endometrium thickness on the day of embryo transfer (mm)	11.33 ± 1.95	11.31 ± 2.04	11.37 ± 2.02	11.23 ± 1.94	0.613	0.069	0.164

^a^Underweight vs. normal weight

^b^Overweight vs. normal weight

^c^Obese vs. normal weight

Data are presented as mean ± SD for continuous variables and *n* (%) for dichotomous variables

Pregnancy outcomes by BMI group are shown in Table 3. In crude analysis, the rates of implantation, clinical pregnancy, miscarriage, and live birth were all similar between underweight and normal-weight groups. However, all outcome parameters were significantly worse in patients with obesity. Implantation rates ranged from 37.5% in the normal BMI category to 35.3% in the BMI ≥ 27.5 kg/m^2^ group (*P* = 0.04), although it failed to demonstrate statistical significance for BMI between 23 and 27.4 kg/m^2^ (36.5%, *P* = 0.092). The miscarriage rate significantly increased with rising BMI from 11.8% in normal-weight women to as high as 18.9% in women with BMI ≥ 27.5 kg/m^2^ (*P* < 0.001). Live birth rate fell from 46.7% in the reference group to 38.3% in women with obesity (*P* < 0.001). To isolate the effect of obesity from PCOS on reproductive outcomes, a subgroup analysis including women without PCOS was performed and we found the same results (Additional file 1: Table S1).

**Table 3:** Reproductive outcomes per embryo transfer

Characteristics	Underweight (< 18.5 kg/m^2^)	*P* value	Normal weight (18.5–22.9 kg/m^2^)	Overweight (23–27.4 kg/m^2^)	*P* value	Obese (≥ 27.5 kg/m^2^)	*P* value
Implantation rate	1738/4790(36.3)		9397/25,076(37.5)	3522/9650(36.5)		806/2283 (35.3)	
OR (95% CI)	0.95(0.89 1.01)	0.119	Reference	0.96(0.91 1.01)	0.092	0.91(0.83 0.99)	0.040
AOR (95% CI)	0.91(0.85 0.96)	0.002	Reference	0.95(0.90 0.99)	0.029	0.80(0.73 0.87)	< 0.001
Biochemical pregnancy rate	1454/2527(57.5)		7855/13,224(59.4)	2959/5079(58.3)		673/1213(55.5)	
OR (95% CI)	0.93(0.86 1.01)	0.081	Reference	0.95(0.89 1.02)	0.160	0.85(0.76 0.96)	0.008
AOR (95% CI)	0.90(0.82 0.98)	0.017	Reference	0.96(0.89 1.02)	0.185	0.83(0.74 0.94)	0.003
Clinical pregnancy rate	1315/2527(52.0)		7106/13,224(53.7)	2671/5079(52.6)		592/1213(48.8)	
OR (95% CI)	0.93(0.86 1.02)	0.117	Reference	0.96(0.90 1.02)	0.164	0.82(0.73 0.92)	0.001
AOR (95% CI)	0.91(0.83 0.99)	0.028	Reference	0.96(0.90 1.02)	0.201	0.81(0.71 0.91)	< 0.001
Multiple pregnancy rate	418/2527(16.5)		2255/13,224(17.1)	839/5079(16.5)		208/1213(17.1)	
OR (95% CI)	0.96(0.86 1.08)	0.531	Reference	0.96(0.88 1.05)	0.389	1.01(0.86 1.18)	0.933
AOR (95% CI)	0.93(0.83 1.05)	0.236	Reference	0.96(0.88 1.05)	0.411	0.99(0.84 1.17)	0.910
Miscarriage rate	147/1315(11.2)		837/7106(11.8)	387/2671(14.5)		112/592(18.9)	
OR (95% CI)	0.94(0.78 1.14)	0.534	Reference	1.27(1.12 1.45)	< 0.001	1.75(1.41 2.17)	< 0.001
AOR (95% CI)	1.03(0.86 1.25)	0.741	Reference	1.21(1.06 1.38)	0.004	1.66(1.32 2.08)	< 0.001
1st trimester	139/1315(10.6)		763/7106(10.7)	332/2671(12.4)		90/592(15.2)	
OR (95% CI)	0.98(0.81 1.19)	0.857	Reference	1.18(1.03 1.35)	0.018	1.49(1.18 1.89)	0.001
AOR (95% CI)	1.07(0.88 1.30)	0.487	Reference	1.14(0.99 1.32)	0.060	1.46(1.15 1.87)	0.002
2nd trimester	8/1315(0.6)		74/7106(1.0)	55/2671(2.1)		22/592(3.7)	
OR (95% CI)	0.58(0.28 1.21)	0.147	Reference	2.00(1.41 2.84)	< 0.001	3.67(2.26 5.95)	< 0.001
AOR (95% CI)	0.65(0.31 1.35)	0.243	Reference	1.74(1.22 2.48)	0.002	2.76 (1.67 4.58)	< 0.001
Ongoing pregnancy rate	1176/2527(46.5)		6343/13,224(48.0)	2339/5079(46.1)		502/1213(41.4)	
OR (95% CI)	0.94(0.87 1.03)	0.188	Reference	0.93(0.87 0.99)	0.020	0.77(0.68 0.86)	< 0.001
AOR (95% CI)	0.90(0.83 0.99)	0.023	Reference	0.93(0.88 0.99)	0.046	0.76(0.67 0.86)	< 0.001
Live birth rate	1149/2527(45.5)		6170/13,224(46.7)	2244/5079(44.2)		465/1213(38.3)	
OR (95% CI)	0.95(0.88 1.04)	0.272	Reference	0.91(0.85 0.97)	0.003	0.71(0.63 0.80)	< 0.001
AOR (95% CI)	0.91(0.83 0.99)	0.034	Reference	0.92(0.86 0.98)	0.013	0.70(0.62 0.80)	< 0.001

Data are presented as *n*/*n* (%) for dichotomous variables

*OR* Odd ratio, *AOR* adjusted odd ratio, Implantation rate was defined as the number of gestational sacs visualized on ultrasound examination divided by the number of embryos transferred. Ongoing pregnancy rate was defined as a viable pregnancy that progressed beyond 12 gestational weeks divided by the number of embryo transfer cycles for each group. Live birth rate was defined as the number of live births divided by the number of embryo transfer cycles for each group

Analyses were adjusted for age, infertility duration, gravidity, parity, main cause of infertility, number of OPU prior to FET, year of treatment, number of embryo transferred, and embryo developmental stage at transfer

After adjustment for a number of confounding factors, however, underweight women were associated with slightly reduced rates of implantation (adjusted odds ratio (aOR) 0.91; 95% CI 0.85–0.96), clinical pregnancy (aOR 0.91; 95% CI 0.83–0.99), and live birth (aOR 0.91; 95% CI 0.83–0.99) whereas the association between pregnancy loss and underweight was insignificant. Compared to the normal-weight controls, obesity women had a significantly decreased chance of implantation (aOR 0.80; 95% CI 0.73–0.87), clinical pregnancy (aOR 0.81; 95% CI 0.71–0.91), and live birth (aOR 0.70; 95% CI 0.62–0.80). Furthermore, the risk of pregnancy loss in the first (aOR 1.46; 95% CI 1.15–1.87) or the second trimester (aOR 2.76; 95% CI 1.67–4.58) or as a whole (aOR 1.66; 95% CI 1.32–2.08) was all significantly higher in obesity women than that in the reference controls. The details of the covariate adjustment in the multiple logistic model for live birth rate are listed in Table 4.

**Table 4:** Results of multiple regression analysis for live birth rates

	OR	95% C.I.	*P* value
Normal weight	Reference		
Underweight	0.91	0.83–0.99	0.034
Overweight	0.92	0.86–0.98	0.013
Obese	0.70	0.62–0.80	< 0.001
Age	0.96	0.96–0.97	< 0.001
Infertility duration	0.97	0.96–0.98	< 0.001
Gravidity	0.96	0.94–0.99	0.005
Parity	0.88	0.79–0.97	0.014
Main cause of infertility			
Tubal factor	Reference		
PCOS	1.12	1.02–1.23	0.018
Diminish ovarian reserve	1.06	0.94–1.19	0.340
Endometriosis	1.07	0.96–1.18	0.230
Uterine factor	0.75	0.64–0.88	< 0.001
Unexplained	1.07	0.91–1.25	0.410
Male factor	1.20	1.11–1.30	< 0.001
Number of OPU prior to FET	0.92	0.88–0.95	< 0.001
Year of treatment	1.09	1.08–1.11	< 0.001
Number of embryo transferred	2.14	1.93–2.37	< 0.001
Embryo developmental stage at transfer	1.81	1.59–2.05	< 0.001

Given that endometrial preparation is an important mediating factor between BMI and pregnancy outcomes, data was separately analyzed according to the type of endometrial priming protocol. As shown in Additional file 1: Table S2, the decrease in live birth rates noted in obesity women remained consistent after stratified analysis.

Perinatal outcomes by BMI categories are shown in Additional file 1: Table S3. The odds of preterm birth and low birth weight were both significantly higher in singletons resulting from obesity group as compared with those from normal-weight controls. Further, women with elevated BMI had increased rates of pregnancy-related complications.

## Discussion

In this large observation study, we investigated the impact of BMI on pregnancy outcomes in women undergoing FET with high-quality embryo transfer. Our results indicated that based on a freeze-all policy, underweight status has limited impact on reproductive outcomes whereas obesity was associated with decreased rates of clinical pregnancy and live birth.

Earlier observation studies failed to find a relationship between adverse reproductive outcomes and increasing BMI [19, 20]. These studies suffered from several drawbacks such as small sample sizes and methodological flaws, making the results difficult for robust conclusions. Of late, a number of large trials improved on the abovementioned flaws, unequivocally showed poor IVF outcomes in obesity women. Analysis of a large dataset from Society for Assisted Reproductive Technology (SART) based on 239,127 fresh autologous cycles revealed progressive and worsened pregnancy outcomes in women with higher BMI as compared with normal-weight peers [21]. Data from the National ART Surveillance System, including more than 400,000 transfer cycles, demonstrated that obesity negatively impacts all ART and obstetric outcomes investigated [22]. The work by Rittenberg and colleagues indicated that BMI of ≥ 25 kg/m^2^ significantly increased the risk of clinical miscarriage before 23 weeks of gestation in both fresh and cryo-thawed cycles [23]. Our study, exclusively focusing on FET cycles, adds further to the current existing literature by suggesting that the detrimental impact of high BMI was not overcome by transfer of embryos into a more physiologic uterine environment.

Contrary to our results, Farhi et al. found that transfer of high-quality embryos overcame the adverse effect of obesity on implantation and pregnancy rates in women referred to IVF treatment [24]. Nonetheless, Farhi simply divided subjects into two groups: BMI ≤ 25 kg/m^2^and BMI > 25 kg/m^2^, without making a distinction between overweight and obesity. In addition, a total of 233 patients were included in that study, of which merely 73 women had BMI > 25 kg/m^2^. A more recent study on FET cycles failed to find an association between BMI and implantation rate among cycles with high-quality blastocyst transfers following identical hormonal endometrial preparation. However, the authors acknowledged that their study was underpowered to detect differences in the underweight and obesity cohorts, with only 8 underweight women and 59 obesity women [14].

Since the relationship between raised BMI and poor pregnancy outcomes has been well-established, the next question to be answered is whether this effect is caused by oocyte/embryo, endometrium, or both. A large retrospective study including more than 45,000 ART cycles reported higher BMI was associated with an increased risk of failure to achieve a clinical intrauterine gestation among women using autologous oocytes; however, this risk was overcome by the use of donor oocytes [25]. These results suggested that oocyte quality, rather than uterine receptivity, might account for the decreased fertility related with increasing BMI. Shah et al. observed that obesity was associated with fewer normally fertilized oocytes, few embryos available, and subsequently lower pregnancy rate, supporting the idea that the detrimental effect of body weight occurs at the ovary level [7]. On the other hand, Bellver et al. analyzed 9587 recipient cycles with normal BMI donors and observed impaired reproductive outcomes as BMI increased, indicating that uterine receptivity seems to be altered in obesity recipients [26]. This concept was further supported by recent evidence that obesity women with recurrent miscarriage are more likely to have euploid embryos, pointing to the endometrium as the main player in obesity-related reproductive impairments [27]. The present study, eliminating the potential influences of obesity on oocyte/embryo quality, again highlighted the undermined endometrial receptivity is to blame, at least partly, for the discouraging IVF results reported in obese patients.

The mechanism by which obesity disturbed endometrial function has yet to be fully elucidated. Obesity-related metabolic disorder, such as altered glucose metabolism and insulin resistance-impaired endometrial receptivity, resulted in implantation failure and recurrent spontaneous abortion [28, 29]. Transcriptomic analysis identified obesity was associated with significantly altered endometrial gene expression during the window of implantation in infertile obesity women as compared with infertile normal-weight controls. Such gene dysregulation may contribute to the poor IVF outcomes observed in obesity patients [30]. A recent preliminary study noticed that endometrial decidualization, a key step for successful implantation and placentation, was impaired in diet-induced obesity mice [31]. A similar phenomenon was also found in endometrial stromal cell isolated from obesity women. The authors provided new insight that decreased decidualization and obesity-related subfertility can be attributed to autophagy defects.

At the other extreme of body weight, being underweight had virtually similar rates of implantation, clinical pregnancy, and miscarriage. Even if the live birth rate was statistically significant after adjustment for potential confounders, the difference was too small to be clinically relevant. Likewise, there were few differences in pregnancy outcomes between normal-weight and overweight groups except for a marginal live birth rate reduction observed in overweight women. However, overweight subjects had increased odds of pregnancy-related complication as compared with normal-weight controls.

It has been suggested that the association between BMI, the percentage of body fat, and body fat distribution differs across populations [32]. Specifically, Asians generally have a higher body fat but a lower BMI than that in white people of the same age and sex [15]. Thus, we used BMI cut-off points based on Asia criteria. Noteworthy was that we had analyzed the data using Caucasian BMI cutoffs and found the same results.

Our study was limited by its retrospective design. In this context, we meticulously screened the database with strict inclusion criteria, and analysis was restricted to first embryo transfer cycles. Additionally, the vast majority of patients in the present study were transferred two cleavage-stage embryos, rather than single blastocyst transfer. Chinese legislation limited the proportion of blastocyst transfer cycles within 7% to control the male birth. Thus, transfer of two cleavage-stage embryos remained a priority in Chinese IVF centers [33, 34]. Fortunately, this policy has been abolished since 2019, and the investigation is underway to look into whether maternal BMI has any influence on reproductive outcomes of women with single blastocyst transfers. The current study also had several strengths. The primary strength was the large cohort size; to the best of our knowledge, this study is the largest to date in this area evaluating the impact of BMI on reproductive outcomes resulting from a freeze-all policy. A number of potential confounders that might otherwise have biased the results were controlled in the current study. Furthermore, all cycle data were derived from a single institution, where practice consistency can be assured. Except for the type of culture medium, all other IVF procedures and laboratory conditions remained unchanged during the study period.

## Conclusions

In summary, our study adds new information to the fields of ATR and BMI research, demonstrating that obesity is detrimental to IVF outcomes. Our finding highlights that the negative impact of excess body weight may occur at the level of endometrium. Future prospective researches are needed to further verify our results.

Women with high BMI not only experience poor pregnancy outcomes but rather have greater risks of adverse obstetric and neonatal complications such as preterm delivery, gestational diabetes, and abnormal neonatal birthweight [3]. Most importantly, these maternal and neonatal complications have long-term health consequences for both the mother and her child [32]. Therefore, clinicians should adequately counsel patients about short- and long-term effects of elevated BMI on pregnancy outcomes and resulting infants and further encourage them to attain normal weight prior to fertility treatment.

## Additional files

Additional file 1: **Table S1.** Reproductive outcomes of patients without PCOS. **Table S2.** Reproductive outcomes stratified by the type of endometrial preparation. **Table S3.** Perinatal outcomes by body mass index. (DOCX 40 kb)

Additional file 2: **Figure S1.** Flow chart of the study. (JPG 2061 kb)

## Data Availability

The datasets used and/or analyzed during the current study are available from the corresponding author on reasonable request.

## Abbreviations

aOR: Adjusted odds ratio
BMI: Body mass index
CI: Confidential interval
FET: Frozen-thawed embryo transfer
IVF: In vitro fertilization
OPU: Ovum pick-up
OR: Odds ratio
