# Supplementary material for: Effect of body mass index on pregnancy outcomes in a freeze-all policy: an analysis of 22,043 first autologous frozen-thawed embryo transfer cycles in China
Source: BMC Med. 2019 Jun 26;17:114. doi: 10.1186/s12916-019-1354-1 (PMC6593528; doi:10.1186/s12916-019-1354-1)
Supplement: Supplementary file 1 — Table S1. Reproductive outcomes of patients without PCOS. Table S2. Reproductive outcomes stratified by the type of endometrial preparation. Table S3. Perinatal outcomes by body mass index. (DOCX 40 kb) [file 12916_2019_1354_MOESM1_ESM.docx]

Additional file 1 Table S1 Reproductive outcomes of patients without PCOS.

| Characteristics | Underweight  (<18.5 kg/m^2^) | Normal weight  (18.5-22.9 kg/m^2^) | Overweight  (23-27.4 kg/m^2^) | Obese  (≥27.5 kg/m^2^) |
| --- | --- | --- | --- | --- |
|  | n=2,365 | n=12,147 | n=4,283 | n=867 |
| Clinical pregnancy rate | 1,221/2,365(51.6) | 6,473/12,147(53.3) | 2,206/4,283(51.5) | 387/867(44.6) |
| Odd ratio (95% CI) | 0.94(0.86 1.02) | Reference | 0.93(0.87 0.99) | 0.71(0.62 0.81) |
| P-value | 0.139 | Reference | 0.044 | <0.001 |
| AOR(95% CI) | 0.91(0.83 0.99) | Reference | 0.96(0.89 1.03) | 0.74(0.64 0.85) |
| P-value | 0.028 | Reference | 0.204 | <0.001 |
| Miscarriage rate | 138/1,221(11.3) | 763/6,473(11.8) | 314/2,206(14.2) | 71/387(18.3) |
| Odd ratio (95% CI) | 0.95(0.79 1.16) | Reference | 1.24(1.08 1.43) | 1.68(1.29 2.20) |
| P-value | 0.629 | Reference | 0.003 | <0.001 |
| AOR(95% CI) | 1.04(0.86 1.27) | Reference | 1.20(1.04 1.38) | 1.62(1.24 2.13) |
| P-value | 0.665 | Reference | 0.015 | <0.001 |
| Live-birth rate | 1,064/2,365(45.0) | 5,624/12,147(46.3) | 1,866/4,283(43.6) | 306/867(35.3) |
| Odd ratio (95% CI) | 0.95(0.87 1.04) | Reference | 0.90(0.84 0.96) | 0.63(0.55 0.73) |
| P-value | 0.242 | Reference | 0.002 | <0.001 |
| AOR(95% CI) | 0.90(0.83 0.99) | Reference | 0.93(0.86 0.99) | 0.66(0.57 0.77) |
| P-value | 0.026 | Reference | 0.032 | <0.001 |

Analyses were adjusted for age, infertility duration, gravidity, parity, number of OPU prior to FET, year of treatment, number of embryo transferred, embryo developmental stage at transfer.

Additional file 1 Table S2 Reproductive outcomes stratified by the type of endometrial preparation.

| Characteristics | Underweight  (<18.5 kg/m^2^) | Normal weight  (18.5-22.9 kg/m^2^) | Overweight  (23-27.4 kg/m^2^) | Obese  (≥27.5 kg/m^2^) |
| --- | --- | --- | --- | --- |
| Modified Natural cycle | n=1,202 | n=6,104 | n=1,961 | n=356 |
| Clinical pregnancy rate | 623/1,202(51.8) | 3,329/6,104(54.5) | 1,039/1,961(53.0) | 175/356(49.2) |
| Odd ratio (95% CI) | 0.90(0.79 1.02) | Reference | 0.94(0.85 1.04) | 0.81(0.65 0.99) |
| P-value | 0.085 | Reference | 0.229 | 0.048 |
| AOR(95% CI) | 0.86(0.76 0.98) | Reference | 0.95(0.86 1.06) | 0.85(0.68 1.05) |
| P-value | 0.023 | Reference | 0.367 | 0.136 |
| Miscarriage rate | 73/623(11.7) | 386/3,329(11.6) | 147/1,039(14.1) | 34/175(19.4) |
| Odd ratio (95% CI) | 1.01(0.78 1.32) | Reference | 1.26(1.02 1.54) | 1.84(1.25 2.71) |
| P-value | 0.930 | Reference | 0.028 | 0.002 |
| AOR(95% CI) | 1.13(0.86 1.47) | Reference | 1.20(0.97 1.47) | 1.68(1.12 2.51) |
| P-value | 0.394 | Reference | 0.094 | 0.011 |
| Live-birth rate | 544/1,202(45.3) | 2,904/6,104(47.6) | 877/1,961(44.7) | 137/356(38.5) |
| Odd ratio (95% CI) | 0.91(0.81 1.03) | Reference | 0.89(0.81 0.99) | 0.69(0.55 0.86) |
| P-value | 0.141 | Reference | 0.028 | 0.001 |
| AOR(95% CI) | 0.86(0.76 0.98) | Reference | 0.92(0.82 1.02) | 0.74(0.59 0.92) |
| P-value | 0.021 | Reference | 0.096 | 0.007 |
| Stimulated cycles | n=655 | n=3,479 | n=1,588 | n=434 |
| Clinical pregnancy rate | 353/655(53.9) | 1,971/3,479(56.7) | 889/1,588(56.0) | 216/434(49.8) |
| Odd ratio (95% CI) | 0.89(0.76 1.06) | Reference | 0.97(0.86 1.10) | 0.76(0.62 0.93) |
| P-value | 0.191 | Reference | 0.655 | 0.007 |
| AOR(95% CI) | 0.89(0.75 1.05) | Reference | 0.99(0.89 1.13) | 0.80(0.65 0.98) |
| P-value | 0.163 | Reference | 0.993 | 0.028 |
| Miscarriage rate | 35/353(9.9) | 202/1,971(10.2) | 113/889(12.7) | 33/216(15.3) |
| Odd ratio (95% CI) | 0.96(0.66 1.41) | Reference | 1.28(0.99 1.63) | 1.58(1.06 2.35) |
| P-value | 0.849 | Reference | 0.052 | 0.025 |
| AOR(95% CI) | 1.03(0.70 1.50) | Reference | 1.23(0.96 1.58) | 1.54(1.02 2.33) |
| P-value | 0.897 | Reference | 0.100 | 0.041 |
| Live-birth rate | 312/655(47.6) | 1,740/3,479(50.0) | 764/1,588(48.1) | 175/434(40.3) |
| Odd ratio (95% CI) | 0.91(0.77 1.07) | Reference | 0.93(0.82 1.04) | 0.68(0.55 0.83) |
| P-value | 0.264 | Reference | 0.209 | <0.001 |
| AOR(95% CI) | 0.90(0.76 1.06) | Reference | 0.94(0.84 1.06) | 0.67(0.55 0.83) |
| P-value | 0.213 | Reference | 0.335 | <0.001 |
| Hormonal replacement | n=670 | n=3,641 | n=1,530 | n=423 |
| Clinical pregnancy rate | 339/670(50.6) | 1,806/3,641(49.6) | 743/1,530(48.6) | 201/423(47.5) |
| Odd ratio (95% CI) | 1.04(0.88 1.23) | Reference | 0.96(0.85 1.08) | 0.92(0.75 1.13) |
| P-value | 0.636 | Reference | 0.495 | 0.417 |
| AOR(95% CI) | 1.00(0.85 1.19) | Reference | 0.93(0.82 1.05) | 0.85(0.69 1.05) |
| P-value | 0.972 | Reference | 0.226 | 0.135 |
| Miscarriage rate | 39/339(11.5) | 249/1,806(13.8) | 127/743(17.1) | 45/201(22.4) |
| Odd ratio (95% CI) | 0.81(0.57 1.16) | Reference | 1.29(1.02 1.63) | 1.80(1.26 2.58) |
| P-value | 0.259 | Reference | 0.033 | 0.001 |
| AOR(95% CI) | 0.89(0.62 1.29) | Reference | 1.22(0.96 1.55) | 1.71(1.18 2.47) |
| P-value | 0.547 | Reference | 0.111 | 0.005 |
| Live-birth rate | 293/670(43.7) | 1,526/3,641(41.9) | 603/1,530(39.4) | 153/423(36.2) |
| Odd ratio (95% CI) | 1.08(0.91 1.27) | Reference | 0.90(0.80 1.02) | 0.79(0.64 0.97) |
| P-value | 0.381 | Reference | 0.096 | 0.023 |
| AOR(95% CI) | 1.02(0.86 1.21) | Reference | 0.89(0.79 1.01) | 0.75(0.60 0.93) |
| P-value | 0.806 | Reference | 0.075 | 0.008 |

Analyses were adjusted for age, infertility duration, gravidity, parity, main cause of infertility, number of OPU prior to FET, year of treatment, number of embryo transferred, embryo developmental stage at transfer.

Additional file 1 Table S3 Perinatal outcomes by body mass index.

| Characteristics | Underweight  (<18.5 kg/m^2^) | Normal weight  (18.5-22.9 kg/m^2^) | Overweight  (23-27.4 kg/m^2^) | Obese  (≥27.5 kg/m^2^) | P-value^a^ | P-value^b^ | P-value^c^ |
| --- | --- | --- | --- | --- | --- | --- | --- |
| Singletons | n=815 | n=4,392 | n=1,606 | n=330 |  |  |  |
| Newborn gender |  |  |  |  | **0.705** | 0.487 | 0.605 |
| Female | 384(47.1) | 2,101(47.8) | 752(46.8) | 153(46.4) |  |  |  |
| Male | 431(52.9) | 2,291(52.2) | 854(53.2) | 177(53.6) |  |  |  |
| Birthweight | 3,269.04±444.85 | 3,338.86±472.55 | 3,402.10±520.07 | 3,410.29±654.34 | <0.001 | <0.001 | 0.010 |
| Preterm birth(<37weeks) | 38/815(4.7) | 256/4,392(5.8) | 108/1,606(6.7) | 40/330(12.1) | **0.185** | 0.198 | <0.001 |
| Low birthweight (<2500g) | 26/815(3.2) | 164/4,392(3.7) | 66/1,606(4.1) | 25/330(7.6) | **0.447** | 0.502 | 0.001 |
| Macrosomia (≥4000g) | 37/815(4.5) | 312/4,392(7.1) | 184/1,606(11.5) | 66/330(20.0) | **0.007** | <0.001 | <0.001 |
| Pregnancy-related complications | 52/815(6.4) | 362/4,392(8.2) | 237/1,606(14.8) | 77/330(23.3) | **0.071** | <0.001 | <0.001 |
| Twins | n=334 | n=1,778 | n=638 | n=135 |  |  |  |
| Newborn gender |  |  |  |  | **0.082** | 0.119 | 0.401 |
| Female | 344(51.5) | 1,701(47.8) | 578(45.3) | 122(45.2) |  |  |  |
| Male | 324(48.5) | 1,855(52.2) | 698(54.7) | 148(54.8) |  |  |  |
| Birthweight | 2,456.51±457.73 | 2,536.06±452.75 | 2,555.56±503.51 | 2,542.80±468.09 | <0.001 | 0.200 | 0.814 |
| Preterm birth(<37weeks) | 163/334(48.8) | 836/1,778(47.0) | 326/638(51.1) | 72/135(53.3) | **0.549** | 0.077 | 0.157 |
| Low birthweight (<2500g) | 314/668(47.0) | 1,347/3,556(37.9) | 460/1,276(36.1) | 104/270(38.5) | <0.001 | 0.247 | 0.835 |
| Pregnancy-related complications | 31/334(9.3) | 200/1,778(11.2) | 118/638(18.5) | 26/135(19.3) | **0.291** | <0.001 | 0.005 |

a Underweight vs. Normal weight

b Overweight vs. Normal weight

c Obese vs. Normal weight
