# Supplementary figures and images for: Effect of body mass index on pregnancy outcomes in a freeze-all policy: an analysis of 22,043 first autologous frozen-thawed embryo transfer cycles in China
Source: BMC Med. 2019 Jun 26;17:114. doi: 10.1186/s12916-019-1354-1 (PMC6593528; doi:10.1186/s12916-019-1354-1)

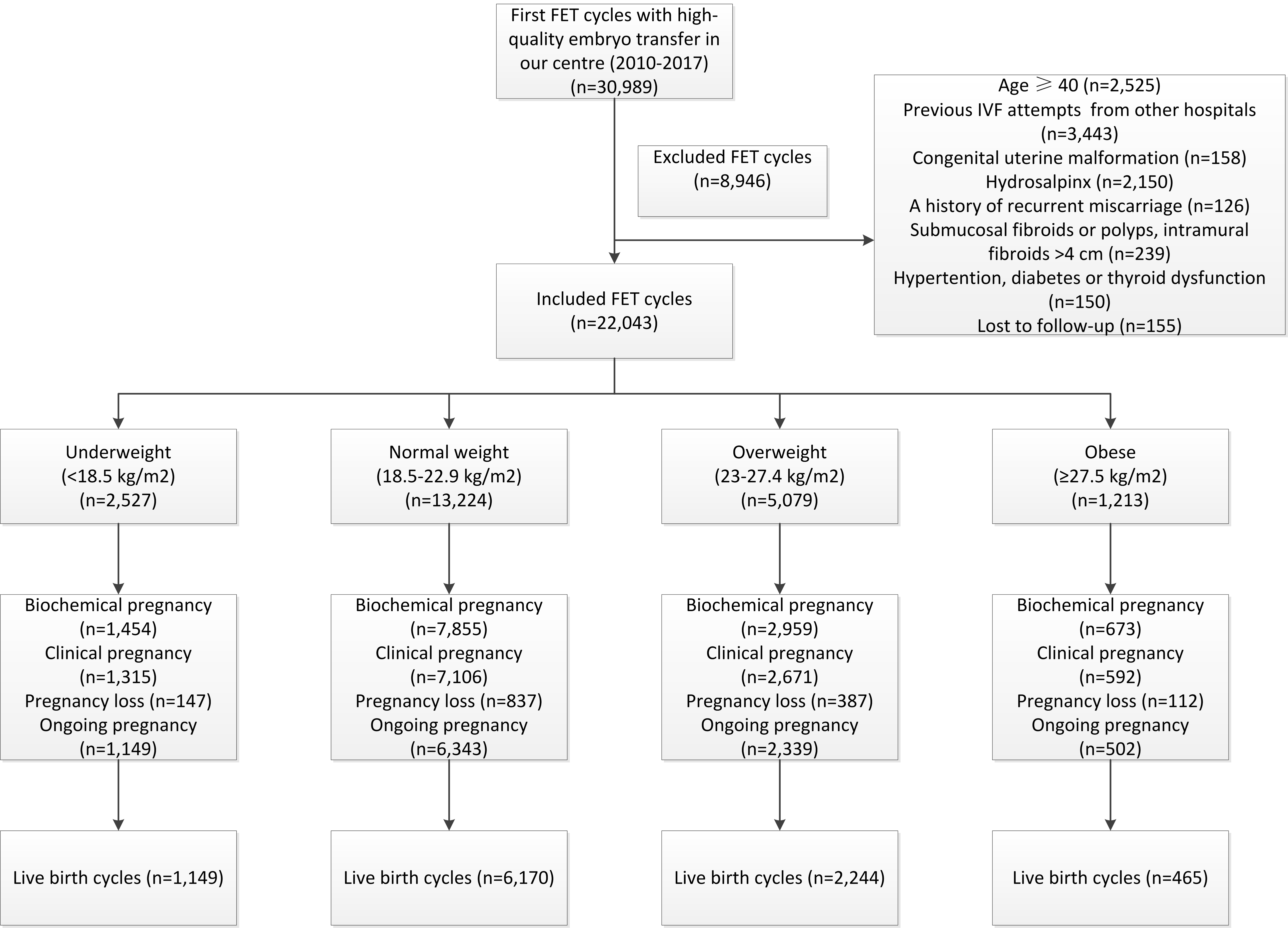

Supplement: Supplementary file 2 — Figure S1. Flow chart of the study. (JPG 2061 kb) [file 12916_2019_1354_MOESM2_ESM.jpg]
